# Pediatric Educational Discussion Scenarios: Reflect, Inspire, Support, and Empower (PEDS-RISE)—A Difficult Patient Encounter Video Scenario

**DOI:** 10.15766/mep_2374-8265.11522

**Published:** 2025-04-30

**Authors:** Celine Payne, Hillary Zieve, Candice Taylor Lucas, Negar Shekarabi, Monisha Vasa, Vara Reddy, Kelly Bauer, Behnoosh Afghani

**Affiliations:** 1 Second-Year Medical Student, University of California, Irvine, School of Medicine; 2 Assistant Professor, Department of Pediatrics, University of California, Irvine, School of Medicine and Children's Hospital of Orange County; 3 Professor, Department of Pediatrics, University of California, Irvine, School of Medicine; 4 Clinical Psychologist and Program Director, Faculty/Staff Support Services, UCI Health Susan Samueli Integrative Health Institute; 5 Clinical Psychiatrist and Volunteer Assistant Clinical Professor, Department of Family Medicine, University of California, Irvine, School of Medicine; 6 Professor, Department of Pediatrics, University of California, Irvine, School of Medicine and Children's Hospital of Orange County

**Keywords:** Difficult Patient, Communication Skills, Pediatrics, Case-Based Learning, Physician-Patient Relationship

## Abstract

**Introduction:**

Difficult patient encounters can lead to burnout and stress in health care workers. Limited training exists to teach residents communication and coping skills needed for dealing with difficult patient encounters.

**Methods:**

We developed a 1-hour virtual video-case scenario workshop to teach communication skills and coping strategies to first-year pediatric residents. The workshop consisted of a short video-case scenario, which was developed in collaboration among ambulatory pediatricians and a hospitalist. The case scenario was followed by guidance from a psychologist and psychiatrist. As part of the workshop, the facilitator provided the residents an opportunity to self-reflect during and after reviewing the case scenario. The effectiveness of the workshop was evaluated using a pre- and post-assessment survey.

**Results:**

All 64 first-year pediatric residents completed the pre- and postworkshop surveys. Before the workshop, only 22% of residents stated familiarity with coping and communication strategies for handling difficult patient encounters. Pre- and postworkshop surveys demonstrated statistically significant increases in the residents’ self-perceived comfort in using strategies to communicate with distressed patients, their ability to identify the need for a debriefing session for themselves or a colleague, and their ability to hold a debriefing session for a junior colleague.

**Discussion:**

Our results suggest that video-case scenarios in combination with facilitated reflection serve as a model to enhance resident training by teaching them skills needed to communicate with patients in difficult situations and helping them cope with distressing events. Such case scenarios may be further applied to other fields of health care.

## Educational Objectives

By the end of this activity, participants will be able to:

1. Describe communication strategies to help with difficult patient encounters.
2. Recognize signs and triggers of distress in self and colleagues dealing with a difficult encounter.
3. Identify when a debriefing session is needed after a difficult patient encounter.
4. Develop skills to hold a debriefing session after a difficult patient encounter.
5. Recognize the available support systems.

## Introduction

Medical residents and physicians commonly confront challenging clinical situations, such as managing difficult patient encounters.^1,2^ These difficult encounters can be overwhelming for medical students and resident trainees as they start their clinical rotations.^2–4^ However, most trainees do not receive formal training on how to approach and cope with these situations.^3,4^ Dealing with difficult patient encounters on a regular basis can lead to burnout, stress, and anxiety.^5^ One way to process these distressing events and mitigate burnout and stress is to teach learners skills in managing difficult patient scenarios and stress through debriefing.^1^ However, training modules for medical residents on improving their communication skills during difficult encounters or holding debriefing sessions are uncommon in medical school or residency programs.^1,2,4,6^

With overall increased evidence about the value of physician wellness, a few training programs have designed workshops to help trainees process the emotional aspects of distressful patient encounters, such as death or adverse events.^7–11^ Resources, including those reported in *MedEdPORTAL,* have mostly focused on peer-to-peer debriefing^10^ and resilience training.^11^ Training programs have taught debriefing skills using role-playing scenarios in small groups; have implemented one-time large-group workshops focusing on debriefing strategies employed after the encounter^8–10^; or have designed trainings on how to improve communication skills using standardized patient encounters.^7^ There is a lack of evidence about workshops that use simulated video-case scenarios to not only teach learners strategies to improve their communication skills during a difficult patient encounter but also provide them resources to deal with their own emotional distress after the encounter.^8^ Additionally, studies have shown that barriers to implementing such workshops include competing clinical responsibilities, which result in low attendance, or implementation of workshops near the end of residency, which limits time for application of skills before becoming an independent physician.^8,10^

Given the need for more training to support resident wellbeing when coping with difficult patient encounters, we developed a virtual workshop to enhance first-year pediatric residents’ skills in communicating with patients and their caregivers during challenging encounters, and to discuss coping strategies with residents for dealing with the stresses related to such encounters.

## Methods

As part of the 4-week ACT (Advocacy, Child Abuse, Community Outreach) rotation for first-year pediatric residents, we conducted a synchronous 1-hour virtual workshop via Zoom to provide the knowledge, confidence, and skills to manage difficult patient encounters and review strategies to cultivate resilience in those circumstances. The workshop was called PEDS-RISE (Pediatric Educational Discussion Scenarios: Reflect, Inspire, Support, and Empower) and was facilitated virtually by the director of the workshop, a pediatric hospitalist (Behnoosh Afghani). The virtual workshop was led by the same facilitator using the facilitator guide (Appendix A), and a different set of two to three interns each month. The workshop was typically implemented during the second or third week of the ACT rotation.

The workshop content was created through collaboration among a team of pediatricians (Behnoosh Afghani, Hillary Zieve, Candice Taylor Lucas, Vara Reddy), a psychologist (Negar Shekarabi), and a psychiatrist (Monisha Vasa). We first created the scripts for each actor participant. The scripts were then reviewed by all team members. The video role plays were developed based on these scripts.

The first video (Appendix B), running just over 12 minutes in duration, consisted of a case about an intern who was feeling stressed and incompetent after a difficult patient encounter in which they were faced with a mother's demands for more antibiotics and diagnostic tests for her child, who seemed to have a viral illness. The second part of the video (Appendix B) portrayed the resident, who remained distressed after the encounter, subsequently deciding to meet with a psychologist. The psychologist (Negar Shekarabi) emphasized the importance of recognizing one's own triggers and the need to use coping strategies.

During the workshop, the facilitator pauses the videos at different time points and encourages open discussion among the participants by posing questions and comments (details in the facilitator guide; Appendix A). The facilitator also introduces a toolkit, the Periodic Table for High Concern Communication (Appendix C, used with permission),^12^ and utilizes it throughout the workshop to guide group discussions. This toolkit is a one-page resource that provides strategies to help health care professionals effectively communicate with patients during challenging situations. Due to the detailed nature of the toolkit, which could not be fully covered during the workshop, a copy was emailed to participants after the workshop, for future reference.

The second video (Appendix D), with a total duration of about 14 minutes, depicted a psychiatrist (Monisha Vasa) who reviewed coping and communication strategies for difficult patient encounters, consisting of the 4 Ds (Appendix E):

• De-escalation: using empathetic phrases and body language that validates the patient's/caregiver's experience
• Deep listening: understanding the patient's/caregiver's concerns by close listening and rephrasing
• Debriefing: communicating the medical provider's feelings and triggers with colleagues
• De-stressing: taking time to relieve burnout and anxiety by practicing self-care

A few days prior to the workshop, the learners (interns) were asked to think about a real-world difficult clinical scenario they had encountered previously, and were invited to keep that scenario in mind while watching the video-case scenario during the virtual meeting. During the workshop, the video was paused at different time points by the facilitator, who prompted an open dialogue that included self-reflection and who led the debriefing. The time line below reflects the suggested duration of time per workshop section:

• 5 minutes: introduction and preworkshop survey
• 12 minutes: case scenario and psychologist (Appendix B)
• 18–22 minutes: interactive discussion among the facilitator and the participants, in which the video (Appendix A) would be paused at different time points and a 2–5–minute discussion would take place each time (total discussion time 18–22 minutes)
• 14 minutes: video of the psychiatrist providing coping and communication strategies (Appendix D)
• 6–7 minutes: interactive discussion among the facilitator and the participants
• 5 minutes: concluding remarks and postworkshop survey

Each month, two or three interns participated in the workshop. By the end of the academic year, almost all interns had participated in the workshop. The workshop typically lasted 1 hour; however, when there were three participants, the workshop lasted a few minutes longer than 1 hour. The facilitator guide (Appendix A) provides more details.

We distributed anonymous pre- and postworkshop surveys (Appendices F and G) to assess the residents’ baseline characteristics and self-perceived skills before and after the workshop. Study data were collected and managed using REDCap (Research Electronic Data Capture) at the University of California, Irvine. The University of California, Irvine School of Medicine Institutional Review Board deemed this project exempt. We created the survey based on similar published survey instruments.^8,10^ The survey underwent multiple rounds of an iterative review process, with multiple rounds of feedback and revision by three pediatricians (Behnoosh Afghani, Hillary Zieve, Candice Taylor Lucas) and a psychologist (Negar Shekarabi). The Cronbach's alpha coefficient for the survey was .94, indicating excellent reliability of the survey instrument.^13^ We employed paired *t* tests to compare the pre- and postworkshop responses. A *p* value of less than or equal to .05 was considered statistically significant.

We also asked the participants to provide a free-text response before and after the workshop to describe any perceived barriers in dealing with difficult patient encounters. Three coders (Celine Payne, Kelly Bauer, Behnoosh Afghani) independently reviewed the free-text responses and classified keywords from each response into different themes that the individual coders believed best represented the underlying dimension of each cluster of keywords.^14^ The coders then reached consensus on the labels assigned. To verify our coding, we used free online word cloud software (WorldClouds.com) to illustrate the frequency of keyword clusters obtained from the free text. However, our review of the open-ended responses was exploratory, and more research needs to be completed before we can draw conclusions on the role these barriers may play in successful implementation.

## Results

A total of 64 first-year pediatric residents (composed of 50 females and 14 males) participated in the workshop in groups of two to three participants per month, with the first workshop starting in August 2022 followed by monthly workshops up to November 2024. All participants completed the pre- and postworkshop surveys. Of the 64 respondents, 95% reported that they had dealt with a difficult patient encounter, and only 22% indicated that they had received any training on dealing with such encounters. Comparing the pre- and postsurvey data (Table 1), we demonstrated statistically significant increases in residents’ self-perceived ability to recognize when they or a colleague are distressed, to identify when a debriefing session is needed for themselves or a colleague, and to feel comfortable holding a debriefing session for a junior colleague, as well as significant increases in residents’ perceived awareness of additional support systems that are in place. Additionally, the survey data showed statistically significant increases in residents’ comfort in communicating with demanding and/or distressed patients/parents, and familiarity with strategies like the 4 Ds (Appendix E) and the Periodic Table for High Concern Communication toolkit (Appendix C).

**Table 1.**
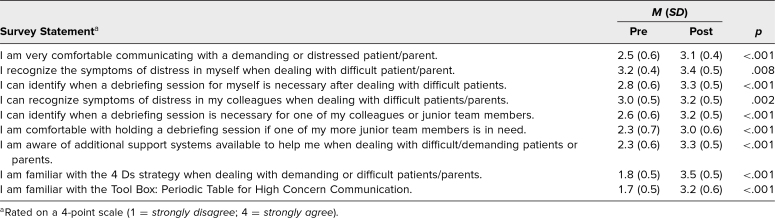
Pre- and Postworkshop Resident Survey Responses (*N* = 64)

### Word Cloud Analysis

**Figure 1.**
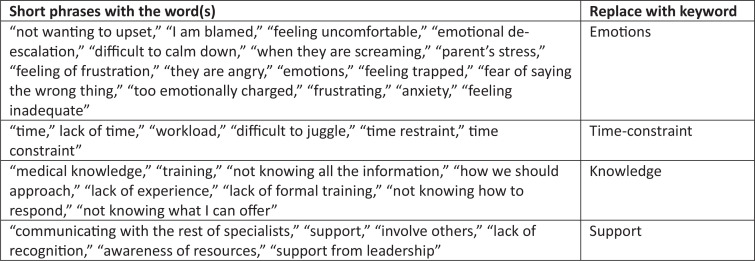
Word cloud replacement keywords.

**Figure 2:**
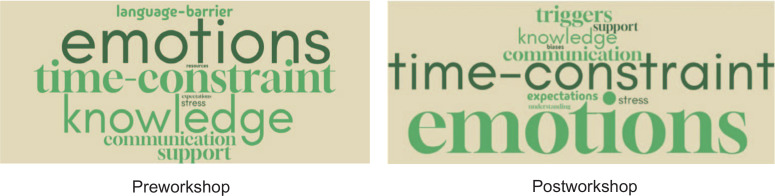
Word clouds of common keywords in the free-text responses to the pre- and postworkshop survey prompt: “Barriers that make it difficult to deal with difficult patient encounters.” Size of text is proportional to the frequency of each keyword. Number of words in word cloud: Preworkshop, emotions = 28, time-constraint = 22, knowledge = 20, support = 8, communication = 7, language-barrier = 6, stress = 4, resources = 2; Postworkshop, emotions = 28, time-constraint = 28, knowledge = 7, communication = 6, triggers = 6, support = 4, stress = 4, expectations = 4.

Synonym keywords were combined manually into a single keyword cluster. For example, phrases that included emotions such as “anger” and “frustration” were grouped as the keyword *emotions*. Similarly, phrases such as “lack of time,” “time,” and “workload” were grouped as the keyword *time-constraint*. A list of the synonym keywords is presented in Figure 1. Using the free online word cloud generator, the coding results were imported into word clouds to illustrate, in a holistic way, the frequency of each item. Figure 2 shows visual representations of the keywords based on responses to the free-text question about barriers in the preworkshop and postworkshop survey. The size of the text in each word cloud is proportional to the frequency of the keyword used in the responses. The most frequent keywords cited as barriers in dealing with difficult patient encounters included *emotions*, *time-constraint*, and *knowledge*.

### Open-Ended Responses

Responses were categorized into themes based on the use of similar or semantically equivalent terms to describe the perceived barriers in dealing with demanding or difficult patients (Table 2). A total of three main themes emerged from both the preworkshop and postworkshop surveys, representing the most common barrier themes, as follows: difficult parental/patient/physician emotions; time constraints; and lack of knowledge/resources/support. Example open-ended comments from respondents pre- and postworkshop are summarized in Table 2.

**Table 2.**
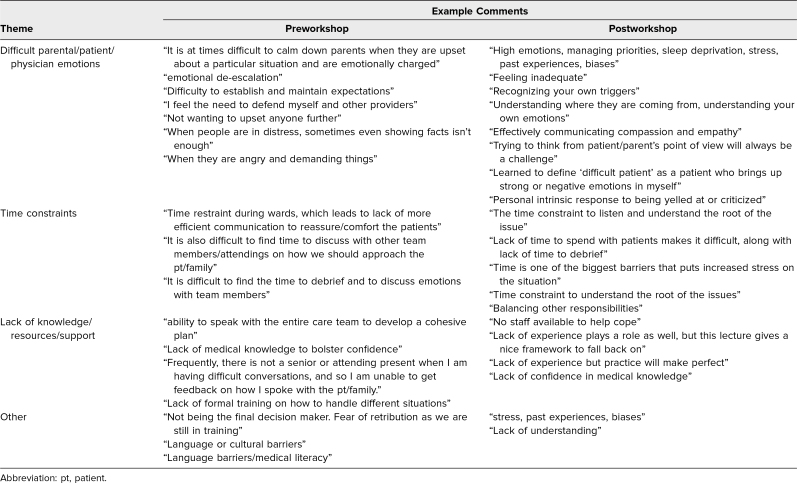
Pre- and Postworkshop Resident Survey Responses to the Prompt: “Please Describe the Barriers That Make It Difficult to Deal With Demanding or Difficult Patients” (*N* = 64)

## Discussion

While more than 95% of our workshop participants had encountered difficult patient interactions, only 22% reported receiving training to manage them. This disparity highlights the urgent need to integrate targeted training into residency programs to boost resident confidence and competence. Overall, our virtual workshop on dealing with difficult patient encounters improved participants’ familiarity with communication and coping skills and ability to recognize symptoms of distress, and enhanced participants’ coping skills through debriefing sessions. While the improvements were statistically significant, there is still potential for further improvement in all areas. Specifically, participants reported that their ability to identify the need for a debriefing session and their skills for holding a debriefing session improved after the workshop, and they became more aware of the additional support systems available to address burnout and persistent distress. Although the participants reported higher self-perceived awareness in recognizing symptoms of distress in themselves or their colleagues after the workshop, this increase was smaller compared to gains in other self-perceived skills. This result may reflect the residents’ difficulty in identifying and regulating emotions, the already high level of stress that residents face in their day-to-day workload, which may prevent them from perceiving additional stress, or possibly a slightly more critical self-lens relative to recognizing distress in a colleague. It is also possible that although the residents observed some of the signs of distress shown in the video-case scenario, they needed more examples of case scenarios portraying the different ways distress can manifest after a difficult encounter. In our future workshops, we plan to expand the examples of different scenarios, where the learners can identify signs of distress in themselves or colleagues, including nonverbal cues through role-plays.^5^

The value of the PEDS-RISE workshop may extend beyond a pediatric residency program. Since the inception of our workshop, other departments in our institution (University of California, Irvine), such as the department of family medicine, have expressed interest in adopting our training workshop for their residents. We believe that several factors have contributed to the success of our workshop. First, we had 100% attendance, whereas attendance was lower in similar workshops reported in previous literature.^8^ We believe that the main reason for the high attendance of our workshop is the support provided by our residency administration in incorporating the workshop into the required ACT curriculum for the interns. In addition, the virtual design of the workshop, as opposed to an in-person workshop, provided easy accessibility from the learner's location of choice. Second, our small-group format offered more opportunities to individualize discussions and provide personalized feedback.^8,10^ Furthermore, implementing the workshop during the intern year provided communication and coping skills early on during residency training. Finally, the interdisciplinary approach among professionals from different specialties, from the project's design to its implementation, was valued by the participants and provided an opportunity for other specialties to reproduce similar video-case scenarios based on specific patient populations.

Barriers to effectively managing difficult patient encounters included handling emotions (of parents, patients, or oneself), time constraints, and a lack of knowledge or support. After the workshop, fewer participants reported a lack of knowledge or resources as a barrier, indicating that our workshop was helpful in enhancing the participant's knowledge about communication and coping strategies. However, handling emotions and time constraints remained a common barrier after the workshop. This finding is unsurprising, as changing management of emotions and addressing time constraints can be challenging after a 1-hour workshop. Comments from some participants, such as “Effectively communicating compassion and empathy” and “Trying to think from the patient/parent's point of view,” suggest that the residents became more aware of the importance of dedicating focused time and demonstrating empathy to communicate effectively with patients and caregivers, while also understanding the root cause of issues in difficult encounters. Lack of time due to heavy patient load can lead to physician fatigue and burnout, which, in turn, can contribute to suboptimal patient care and lower patient satisfaction; the impact of this may be circular, resulting in a lack of any progress in handling the difficult encounter, physician burnout, and well-being.^15,16^ Additional workshops should emphasize strategies for effectively addressing the time constraints faced by residents. For example, since the patient or caretaker may be feeling overwhelmed with information and anxious, communication skills and comfort could be improved through role-plays of breaking the patient–doctor discussion into smaller sessions and providing appropriate feedback. It is important that the trainees show genuine concern during such patient or caretaker encounters; by building a trusting physician–patient relationship, trainees may overcome difficult emotions.

More studies are needed to identify and address the underlying causes of burnout.1,15 Our preliminary study showed that our workshop was effective in enhancing awareness and knowledge about communication and coping strategies. However, to maximize the effect of this workshop, institutions must mitigate the general risk of resident burnout and the specific risk created by managing difficult patient encounters. Institutions have provided some opportunities via the availability of counseling, on-site workout/yoga, green spaces, etc., but more systemic interventions are needed to address critical barriers in dealing with difficult patient encounters, such as time constraints and emotional distress.

In the postworkshop survey, some participants cited barriers such as “recognizing your own triggers” or “personal intrinsic response to being yelled at or criticized.” These comments highlight a key point from the psychologist's debriefing: understanding that one's own triggers, shaped by past experiences, is crucial for coping after challenging encounters. Other participant responses, such as “high emotions, managing priorities, sleep deprivation, stress, past experiences, biases” and “managing high emotions,” suggest that participants may have encountered difficulties with families who did not initially respond to de-escalation efforts. During the live self-reflection, several residents shared their frustration at not seeing improvement despite their efforts to empathize with the patient. In future workshops, we will focus on tackling challenging case scenarios that defy typical communication and coping strategies, especially when time is limited.

Our study is limited by its brevity (1-hour duration), small sample size (*N* = 64), single-center design, and response bias. For example, using pre- and postsurvey responses before and right after the course of the training possibly introduced response bias, with participants overreporting changes in their responses. The short duration between pre- and postworkshop surveys did not allow for assessment of implementation, sustained change, and retained knowledge and skills from the workshop. Our analysis of open-ended responses may also include unmeasured bias. Future directions for this study include assessing communication skills and debriefing by residents in different case scenarios as well as measuring the effectiveness of our workshop by doing additional follow-up surveys to measure its long-term impact. Finally, we realize that a psychologist or psychiatrist may not be readily available for those in need of support; however, we believe that the guidance provided by the psychologist and psychiatrist in this workshop could be used to remind trainees about the coping mechanisms and communication strategies that might be applied when a trainee or their colleague faces a difficult patient encounter. For example, without seeing a psychologist, a trainee or practicing physician might be reminded that being aware of the occurrences that are triggering, as well as understanding the deeper feelings that are being triggered, could help the individual manage their emotions. This advice could also be given to a colleague who is in distress after a difficult patient encounter.

In summary, our virtual workshop, including a video-case scenario with facilitated reflection, could serve as a model to augment resident training by raising awareness and introducing skills to cope and care when faced with difficult patient encounters. Future directions include incorporating more interactive, challenging case scenarios with related role-plays throughout the course of a trainee's residency, and evaluating long-term educational outcomes. Given that all medical providers face similar challenges, these video-case scenarios may be further adapted and applied to other fields of health care and levels of training to enhance the health care learners’ resilience and well-being.

## Appendices


Facilitator Guide.docxDifficult Patient Encounter Scenario.mp4Periodic Table for High Concern Communication.pdfDifficult Patient Psychiatrist Debrief.mp4Summary Slide of 4Ds.pptxPreworkshop Survey.docxPostworkshop Survey.docx
*All appendices are peer reviewed as integral parts of the Original Publication.*
