# Supplementary material for: Pediatric Educational Discussion Scenarios: Reflect, Inspire, Support, and Empower (PEDS-RISE)—A Difficult Patient Encounter Video Scenario
Source: MedEdPORTAL. 2025 Apr 30;21:11522. doi: 10.15766/mep_2374-8265.11522 (PMC12041301; doi:10.15766/mep_2374-8265.11522)
Supplement: Supplementary file 1 — Facilitator Guide.docxDifficult Patient Encounter Scenario.mp4Periodic Table for High Concern Communication.pdfDifficult Patient Psychiatrist Debrief.mp4Summary Slide of 4Ds.pptxPreworkshop Survey.docxPostworkshop Survey.docx [file mep_2374-8265.11522-s001.zip › A. Facilitator Guide.docx]

**Appendix A: Facilitator Guide**

Tips to the facilitator:

1. Remind the participants that the workshop is a safe space and the discussions are confidential
2. Remind the participants about the learning objectives throughout the dialogue
3. Encourage active dialogue by asking questions
4. Facilitator to use personal experiences to encourage an open dialogue
5. Demonstrate compassion and honesty in your discussions
6. Offer support and the option to speak offline
7. Typically, the workshop takes about 1 hours with 2 intern participants but may take a bit longer with 3 participants

**Difficult patient encounter facilitator’s debriefing guide (Appendix B): Patient encounter and the psychologist visit**

| **Time** | **Introduction, reflection and debriefing by the facilitator** | **Facilitator’s debriefing comments about holding debriefing sessions for a colleague in distress. These comments are made after the comments in the previous column.** |
| --- | --- | --- |
| First 2 minutes  Introduction and objectives for the workshop | Hello, my name is (Name of facilitator) and I am a (facilitator’s title and work description). Although our job as physicians is very satisfying, like any other job we sometimes face challenging situations. The (name of institution) residency program has developed a case scenario to help you with some strategies to navigate through some challenging situations. This is a safe space and our discussions will remain confidential.  We realize that not every case is the same and people’s reactions can be different in every case. As I had emailed you before this workshop, when you watch this case scenario, please take a moment to recall similar situations that you have experienced and remember your own feelings, actions, and decisions. We hope that these simulated scenarios help you explore your feelings, values and judgements and support you during similar scenarios in the future.  The objectives of this workshop include:   1. Describe communication strategies to help with difficult patient encounters. 2. Recognize signs and triggers of distress in self and colleagues dealing with a difficult encounter. 3. Identify when a debriefing session is needed after a difficult patient encounter. 4. Develop skills to hold a debriefing session after a difficult patient encounter. 5. Recognize available support systems. |  |
| Next 5 minutes | Ask the participants to complete the voluntary anonymous pre-workshop survey. Tell them that the responses will be put together and may be used for research. |  |
| Start the video  Stop the Video at 1:20 minute mark (Discussion for 3-4 minutes) | In my email before this workshop, I had asked if you could think of a difficult patient encounter that you or one of your colleagues have gone through.  This is a safe space. Can each of you briefly tell me about a difficult patient encounter? | Another goal of our survey is for you to be able to hold a debriefing session for a colleague who is bothered because of a difficult encounter. If your colleague is challenged by a difficult situation, make sure you find a private space to talk and assure them that it is a safe space. |
| Stop video at the 1:57 minute mark  Discussion: 3 minutes | Ask the workshop participants: In this case that the actor resident is frustrated, what is the attending trying to do? Wait for the workshop participants’ responses. Emphasize that the attending is trying to validate the resident’s feeling of frustration.  Ask the workshop participants: Repeat their own experiences about the cases that the participant mentioned as their own experience and ask how would you approach your case differently? What were some of the emotions they went through?  Through this approach, the participants know the importance of being sensitive to their own feeling and recognizing them in others and then taking appropriate action.  What are some phrases that you used or could have used?  Depending on what the workshop participants say, the facilitator can give examples of phrases that convey empathy:  It must be frustrating…  Tell me about your frustration…  I can see that you are upset…  Ask the workshop participants about some other patient feelings that they have seen/experienced: anger, sadness, frustrations, demanding, etc. | This is very important when your colleagues experience similar situations. Consider sharing some of your own experiences to support them and demonstrate your empathy.  Facilitator asks the participants: What do you do when you get distressed? Have you noticed signs of distress in colleagues? What did it look like?  Examples given typically include “being quiet”, “not able to concentrate”, “feeling of not belonging”, “being short with people”  Remembering your own experiences, communicate some of these phrases for conveying empathy to your colleague in a safe space. |
| Stop video at the 4:34 minute mark  Discussion: 3 minutes | Ask the workshop participants: what they thought about the physician-parent interaction? Ask if they have any positive or negative feedback and what kind of body language or interaction may help during a difficult patient encounter.  Most participants will have comments about body language. The facilitator summarizes the comments and adds her/his own suggestions as needed.   - Taking the time and not rushing when dealing with a difficult patient encounter, so the patient knows the physician cares - Sitting down at the same level as the parent/patient with good eye contact. The facilitator expresses that there may not be room to sit down specially in the inpatient setting. It is ok to sit at a corner of the patient’s or caretaker’s bed. If the caretaker is sitting and there is no space for the physician to sit, it is ok to kneel for a few seconds while talking or listening. - Sometimes you have to negotiate with the patient, especially if they are demanding extra diagnostic tests that may not be necessary - Ask the patient for their opinion on how to navigate the conflict and what they feel comfortable with - Do not overwhelm the patient/parent with too much information - Always say what the next step is and that you are going to follow-up | Remember your own experience during a patient encounter and communicate with your colleague about the importance of body language and not rushing when talking to the caretakers.  You can model these behaviors that we have reflected upon during difficult encounters in presence of more junior trainees |
| Stop video at 5:30 minute mark  2 minutes | Facilitator expresses that the emotions that the resident feels during the patient encounter is normal but if those emotions get prolonged to the point that they interfere with the daily function, the resident should get help.  Facilitator also asks if they are aware of the support systems available at the organization and introduces the participants to some of the available support systems.  In the next part of the video case, the resident seeks help from the psychologist.  If a psychologist is not available, the resident should talk to their attending physician. If that is not possible, they can contact their medical education administrator or human resources for wellbeing help. Many hospitals and academic centers have wellbeing resources. | If you recognize a colleague is having a hard time concentrating after a difficult patient encounter, you can support them using the strategies described in this scenario. If the problem persists, you can tell them how they can reach a mental health professional or contact their medical education administration for help. Many schools and hospitals have resources for support. |
| Stop video at the 6:34 minute mark  Discussion: 3-4 minutes | Facilitator asks the participants about how they perceive the conversation so far. The  The facilitator emphasizes that the psychologist is trying to raise awareness about the resident actor’s emotions in the situation.  Facilitator asks the participants if they could remember how they felt during their difficult patient encounter. The participants reflect on their emotions during their own difficult patient encounter.  The facilitator acknowledges the feelings and emotions and gives an example of how she/he felt during a difficult encounter that a parent was asking for narcotics because she thought that her child was suffering from abdominal pain while on the exam there was no evidence of pain. The facilitator explained steps: 1) empathize with the parent’s feeling and express that you are aware that they did not want their child to suffer, 2) explain some of the objective data that did not point toward pain (such as physical exam and vital signs) as well as the side effects of narcotics and 3) discuss some future steps to assure that similar medications will be ordered as needed for pain and the condition will be re-evaluated. | You should ask your colleague about their feelings and emotions.  Always acknowledge the emotions and don’t say they should not feel the way they do.  Normalize the emotions they are going through is difficult. |
| Stop the video at the 9:00 minute mark  Discussion: 2 minutes | Facilitator emphasizes the need to identify the triggers of emotions in both them and the caretaker/patient and establish trust in the first few seconds of conversation by using phrases that convey empathy.  The facilitator introduces and shares the Toolkit: “High Concern Communication” (Appendix D) and reflects on the video case scenario.   1. CCO (compassion, conviction, optimism): “I am sorry to hear about that…”, “I am confident that…” and “In the future, I believe that…”   In this case scenario, the attending physician showed compassion and understanding of the parent’s frustration, she explained the reasoning and showed confidence in her judgement and reassured that she will follow-up with the parent regarding her daughter to see how things are going.   1. VCD (Voice, Choice, Do), Voice: communicating, listening, participation, Choice: Give them options, Do: give them things to do, feeling of hope.   In the case scenario, the physician listened, gave options and gave a feeling of hope that the patient will most likely get better and if not, there will be additional steps taken. | The facilitator reminds the participants to talk about these communication skills to other colleagues who are going through difficult encounters. |
| Stop video at 9:50 mark  Discussion: 1-2 minutes | Facilitator asks the participants, what kind of patients behaviors are triggering for you?  Some examples that the participants mentioned:  Yelling, demanding, guarded, controlling, crying, talking over me, communicating in a busy room, etc.  The facilitator expresses that taking a moment to become aware of their emotions and the triggers and developing ways to manage them can help face challenges in stressful situations.  The facilitator asks what are some ways that work for you to regulate your emotions when triggered. | Facilitator reminds the participants to ask their colleagues in difficult encounters about their emotions and ask them to identify their emotions and triggering events. Although we are not psychologist, encouraging colleagues to become aware of their emotions and triggers will be helpful in regulating their response when triggered. The awareness and regulation will be helpful in coping with difficult patient encounters. |
| End of the video  Discussion: 2 mins | Facilitator shows the High Concern Toolkit (Appendix D) again and reflects on an example of a patient’s caretaker who was overwhelmed with the information about her child’s new diagnosis and refused care. The facilitator goes over a few more important strategies in dealing with such difficult encounters:  R3, Rule of 3: Provide no more than three new messages, ideas or important points  AAF: Acknowledge uncertainty, State what Actions you have taken, provide what will be the Follow-up  KDG: Know, Do, Go: Share what is the most important for people to Know, what is the most important thing to Do, Where can they to Go to get credible information  The facilitator emphasizes that the psychologist is available for the residents if they are having difficulty coping with a patient encounter. The facilitator also shares some of the wellbeing resources that are available at the medical school and hospital. | In helping your colleagues who are dealing with a difficult encounter, you may not have this toolkit (Appendix D) handy but try to remember some of the key strategies. |

**Difficult patient encounter facilitator’s debriefing guide (Appendix C): Coping and communication strategies by the psychiatrist**

| **Time** | **The facilitator’s debriefing and reflection comments** | **Facilitator’s debriefing comments about holding debriefing sessions for a colleague in distress** |
| --- | --- | --- |
| Before the video starts | For the next few minutes one of our psychiatrists will talk about coping strategies and communication skills when dealing with a difficult patient encounter | Please remember these strategies to use them when holding a debriefing with your colleague. |
| Stop video at 4:00 mark  1 minute | The facilitator emphasizes the psychiatrist’s comment about trying to see the situation from the eyes of the patient or the caretaker when dealing with a difficult encounter. For example, realizing that the patient and caretaker are in a vulnerable position, and maybe confused or have other struggles.  The facilitator uses another example of her own experience. “I admitted a teenager with persistent high fevers. The mother appeared “demanding” to the staff and needed to know everything in detail. When I asked the mother: are you doing ok? She responded that one of her family members died in the PICU a couple of weeks ago.” The facilitator expresses that information helped her understand where the mother was coming from.  The facilitator emphasizes that if we don’t know the struggles and some of the background information in a difficult patient encounter, the situation may get more difficult for us to deal with also. | The facilitator guides the participants to make sure your colleagues empathize with patients and caretakers in a difficult encounter using some of the phrases discussed in the workshop and their own previous experiences. |
| Stop video 8:00 mark  2 minutes | The facilitator asks “what are the first 2 D’s so far”? The facilitator emphasizes the importance of  De-escalation “I can see why you are…”  Deep listening: Listening and saying back what they (the caretaker/patient) said to you”  The facilitator stresses that when the emotions are “running high” per the psychiatrist, it can be a struggle to provide the best care. Therefore, de-escalation and deep listening may help the situation. | The facilitator asks: suppose one of your colleagues is asking for your advice about a parent yelling after the colleague walks into a patient’s room.  What advice would you provide your colleague using the 2 D’s?  Examples of phrases for de-escalation are: “I can see that you are upset…”  “It must be frustrating…”  Once the patient or caretaker opens up, you can deeply listen and repeat what they said: “It seems like you are upset because…” |
| Stop video at 14:00 mark  2 minutes | The facilitator goes over the last two D’s (Debriefing with colleagues and De-stressing) and asks the participants if they have a support system at work or at home that they can talk to.  The facilitator emphasizes the importance of peer support and provides other resources such as the toolkit (Appendix D) and the contact information for the psychologist, psychiatrist, and the wellbeing resources available at the medical center. | The facilitator asks the participants if they can recognize signs of distress in their colleagues when dealing with a difficult patient situation. After listening to the participants, the facilitator gives examples of signs to look for.  The facilitator emphasizes looking for signs of distress in their colleagues and providing support to those who are dealing with a difficult patient encounter. |
| After the completion of videos  2 minutes concluding remarks  5 minutes Post-workshop survey | The facilitator asks if the workshop was useful and ways to improve the workshop. The facilitator reminds the participants to write any feedback in the postworkshop survey.  After the participants complete the survey, the facilitator asks the participants to summarize the 4 D’s again. The facilitator emails the participants a slide (Appendix E) that summarizes the 4 D’s and the toolkit (Appendix D) to be used for future reference. |  |
