# Supplementary material for: Pediatric Educational Discussion Scenarios: Reflect, Inspire, Support, and Empower (PEDS-RISE)—A Difficult Patient Encounter Video Scenario
Source: MedEdPORTAL. 2025 Apr 30;21:11522. doi: 10.15766/mep_2374-8265.11522 (PMC12041301; doi:10.15766/mep_2374-8265.11522)
Supplement: Supplementary file 1 — Facilitator Guide.docxDifficult Patient Encounter Scenario.mp4Periodic Table for High Concern Communication.pdfDifficult Patient Psychiatrist Debrief.mp4Summary Slide of 4Ds.pptxPreworkshop Survey.docxPostworkshop Survey.docx [file mep_2374-8265.11522-s001.zip › G. Postworkshop Survey.docx]

Post-Workshop Feedback Survey: Difficult Case Scenarios

This anonymous survey that will take about 5 minutes to complete, will help us understand your challenges in dealing and coping in difficult clinical situations and it will provide us feedback on the effectiveness of our workshop.

**Post-Workshop Feedback Scenario: Dealing with difficult or demanding patients/parents**

Strongly Disagree Disagree Agree Strongly Agree

I am comfortable communicating
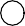

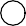

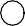

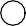
 with a demanding or distressed

patient/parent.

I recognize the symptoms of
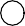

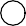

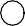

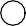
 distress in myself when dealing

with difficult patient/parent.

I can identify when a debriefing
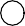

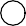

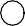

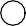
 session for myself is necessary

after dealing with difficult patients.

I can recognize symptoms of
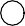

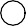

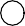

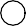
 distress in my colleagues when

dealing with difficult patients/parents.

I can identify when a debriefing
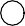

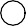

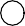

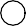
 session is necessary for one of

my colleagues or junior team members.

I am comfortable with holding a
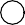

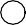

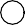

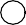
 debriefing session if one of my

more junior team members is in need.

I am aware of the additional
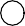

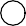

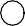

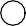
 support systems available to

help me when dealing with difficult/demanding patients or parents.

I am familiar with the 4 Ds
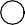

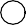

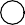

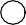
 strategy when dealing with

demanding or difficult patients/parents.

I am familiar with the Tool Box:
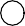

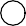

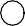

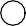
 Periodic Table for High Concern

Communication.

I found this workshop helpful in
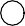

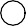

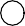

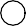
 dealing with demanding or

difficult patients/parents.

Please describe the barriers that make it difficult to deal with demanding or difficult patients?
