# Supplementary material for: Pediatric Educational Discussion Scenarios: Reflect, Inspire, Support, and Empower (PEDS-RISE)—A Difficult Patient Encounter Video Scenario
Source: MedEdPORTAL. 2025 Apr 30;21:11522. doi: 10.15766/mep_2374-8265.11522 (PMC12041301; doi:10.15766/mep_2374-8265.11522)
Supplement: Supplementary file 1 — Facilitator Guide.docxDifficult Patient Encounter Scenario.mp4Periodic Table for High Concern Communication.pdfDifficult Patient Psychiatrist Debrief.mp4Summary Slide of 4Ds.pptxPreworkshop Survey.docxPostworkshop Survey.docx [file mep_2374-8265.11522-s001.zip › F. Preworkshop Survey.docx]

Pre-Workshop Feedback Survey: Difficult Case Scenarios

This anonymous survey that will take about 5 minutes to complete, will help us understand your challenges in dealing and coping in difficult clinical situations and it will provide us feedback on the effectiveness of our workshop.

**Scenario one: Dealing with difficult or demanding patients/parents**

In the past 12 months, have you encountered a Yes situation where you were challenged or distressed by a No difficult or demanding patient/parent?

In the past 12 months, have you been involved in a Yes

debriefing session regarding a difficult or demanding No patient?


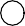

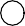

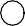

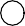

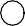

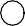


In the past 12 months, have you received any training Yes about dealing with difficult patient or parents? No

Please explain the training you received previously:

**Dealing with a difficult patient/parent, please rank the following:**

Strongly Disagree Disagree Agree Strongly Agree

I am very comfortable
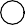

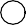

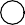

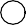
 communicating with a

demanding or distressed patient/parent.

I recognize the symptoms of
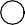

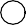

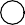

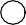
 distress in myself when dealing

with difficult patient/parent.

I can identify when a debriefing
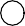

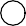

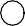

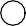
 session for myself is necessary

after dealing with difficult patients.

I can recognize symptoms of
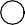

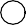

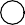

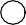
 distress in my colleagues when

dealing with difficult patients/parents.

I can identify when a debriefing
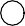

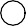

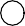

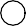
 session is necessary for one of

my colleagues or junior team members.

I am comfortable with holding a
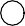

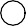

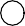

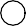
 debriefing session if one of my

more junior team members is in need.

I am aware of the additional
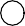

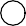

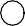

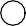
 support systems available to

help me when dealing with difficult/demanding patients or parents.

I am familiar with the 4 Ds
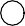

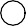

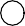

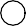
 strategy when dealing with

demanding or difficult patients/parents.

I am familiar with the Tool Box:
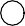

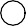

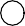

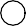
 Periodic Table for High Concern

Communication.

Please describe the barriers that make it difficult to deal with demanding or difficult patients?
