# Supplementary material for: Pediatric Educational Discussion Scenarios: Reflect, Inspire, Support, and Empower (PEDS-RISE)—A Difficult Patient Encounter Video Scenario
Source: MedEdPORTAL. 2025 Apr 30;21:11522. doi: 10.15766/mep_2374-8265.11522 (PMC12041301; doi:10.15766/mep_2374-8265.11522)
Supplement: Supplementary file 1 — Facilitator Guide.docxDifficult Patient Encounter Scenario.mp4Periodic Table for High Concern Communication.pdfDifficult Patient Psychiatrist Debrief.mp4Summary Slide of 4Ds.pptxPreworkshop Survey.docxPostworkshop Survey.docx [file mep_2374-8265.11522-s001.zip › C. Periodic Table for High Concern Communication.pdf]

INSTITUTE for HIGH CONCERN COMMUNICATION

PERIODIC TABLE FOR HIGH CONCERN COMMUNICATION

Use these templates for high concern, risk, crisis, and change management situations

Basic Templates — *Key Templates* — *Advanced Templates*

R3
(Rule of 3)

Use when responding to any high stress or emotionally charged question.

Recommendation: Provide no more than three messages, ideas, or points at a time.

IDK
(I Don't Know)

Use when you don't know, can't answer, or aren't the best source.

Steps:
- Repeat the question (without negatives)
- Say "My ability to answer is limited by...;" or "I don't know"
- Say why you can't answer
- Provide a follow up with a deadline
- Bridge to what you can say

CCO
(Compassion, Conviction, Optimism)

Use when asked a question with high emotion.

Steps:
- Compassion (Caring, Empathy, Listening)
- Conviction
- Optimism

Example: (1) "I am very sorry to hear about...; (2) I'm confident that...; (3) In the future, I believe that..."

ALE
(Authority, Logic, Emotion)

Use to encourage appropriate attitudes, beliefs, or behaviors.

- (A)uthority Message: Appeal to authority-those perceived as high in credibility
- (L)ogic Message: Appeal to logic (if x, then y).
- (E)motion Message: Appeal to an emotion (anger, fear, joy, empathy, surprise, grief, hope, etc).

KDG
(Know, Do, Go)

Use to give upset people a greater sense of control.

- (K)now Message: Share what is most important for people to know.
- (D)o Message: Share what is most important for people to do.
- (G)o Message: Share where people should go for credible information.

P/R
(Primacy/Recency)

Use when responding to any high stress or emotionally charged question.

Recommendation: Provide the most important items or points first and last.

FA
(False Allegation)

Use when responding to a hostile question, false allegation, or criticism.

Steps:
- Repeat/paraphrase question without repeating the negative; repeat underlying value or concern, or use more neutral language
- Indicate the issue is important
- Indicate what you have done, are doing, or will do to address the issue

27/9/3
(27 Words, 9 Seconds, 3 Messages)

Use when responding to any high stress or emotionally charged question.

Recommendation: Be brief and concise in your first response; no more than 27 words, 9 seconds, 3 messages.

TBC
(Trust, Benefit, Control)

Use when responding to questions or concerns indicating high perceived risks or outrage.

- (T)rust Message: Listening to messages communicating listening, caring, or transparency.
- (B)enefit Message: Messages communicating benefits to the individual, org, or society.
- (C)ontrol Message: Messages that give people things to do or give them a sense of control.

KDD
(Know, Do, Do)

Use to give upset people a greater sense of control.

- (K)now Message: Share what is most important for people to know.
- (D)o Message: Share what you are doing to address the concern.
- (D)o Message: Share what people can do to address the concern.

G/WI
(Guarantee/What If)

Used when asked a "what if" question or to guarantee an event or outcome.

Steps:
- Indicate that the question is about the future
- Indicate that the past and the present help predict the future
- Bridge to "what is": known facts, processes or actions

AGL-4
(Average Grade Level Minus Four)

Use when responding to any high stress or emotionally charged question.

Steps: Provide information at four or more grade levels below the average grade level of the audience.

IN=3P
(One Negative Equals Three Positives)

Use when breaking bad news or stating a negative.

Recommendation: Balance one bad news or negative message with at least three or more positive, constructive, or solution-oriented messages.

CAP
(Caring, Action, Perspective)

Use to give upset people a greater sense of control.

- (C)aring Message: Communicates listening, caring, empathy, and compassion.
- (A)ction Message: Actions you are taking to address the concern.
- (P)erspective Message: Helps put the concern in perspective.

VCD
(Voice, Choice, Do)

Use to give upset people a greater sense of control.

- (V)oice Message: Messages communicating listening, dialogue, or participation.
- (C)hoice Message: Messages communicating options, alternatives, or available choices.
- (D)o Message: Messages that give people things to do, increase feelings of hope, etc.

AAF
(Acknowledge, Action, Follow-up)

Steps:
- (A)cknowledge Uncertainty: Identify knowledge gaps and challenges
- (A)ction: State actions you have taken, are taking or will take to address the issue
- (F)ollow-up: Provide information on where people can obtain timely and credible information

Y/N
(Yes/No Template)

Use when asked a yes/no question that cannot be answered yes or no.

Steps:
- Indicate you have been asked yes/no question
- Indicate it would be difficult to answer the question yes or no
- Indicate why it would be difficult to answer the question yes or no
- Respond to the underlying concern

KDK
(Know/Don't Know)

Use when there is high uncertainty.

Steps:
- State what you know
- State what you don't know
- State what you are doing to achieve greater certainty or knowledge

C/S
(Caring/Sharing)

Use when responding to a question or statement containing incorrect information.

- (C)aring Message: State what you and the person holding incorrect information have in common.
- (S)haring Message (1): Invite person holding incorrect information to share their information w/ you.
- (S)haring Message (2): Share the correct information again.

Reference

INSTITUTE for HIGH CONCERN COMMUNICATION

Dr. Vincent Covello, Director
www.centerforriskcommunication.com

MAGA DESIGN

Maga Design, Visual Strategists
www.magadesign.com

© 2014
