# Supplementary material for: Pediatric Educational Discussion Scenarios: Reflect, Inspire, Support, and Empower (PEDS-RISE)—A Difficult Patient Encounter Video Scenario
Source: MedEdPORTAL. 2025 Apr 30;21:11522. doi: 10.15766/mep_2374-8265.11522 (PMC12041301; doi:10.15766/mep_2374-8265.11522)
Supplement: Supplementary file 1 — Facilitator Guide.docxDifficult Patient Encounter Scenario.mp4Periodic Table for High Concern Communication.pdfDifficult Patient Psychiatrist Debrief.mp4Summary Slide of 4Ds.pptxPreworkshop Survey.docxPostworkshop Survey.docx [file mep_2374-8265.11522-s001.zip › E. Summary Slide of 4Ds.pptx]

## Slide 1
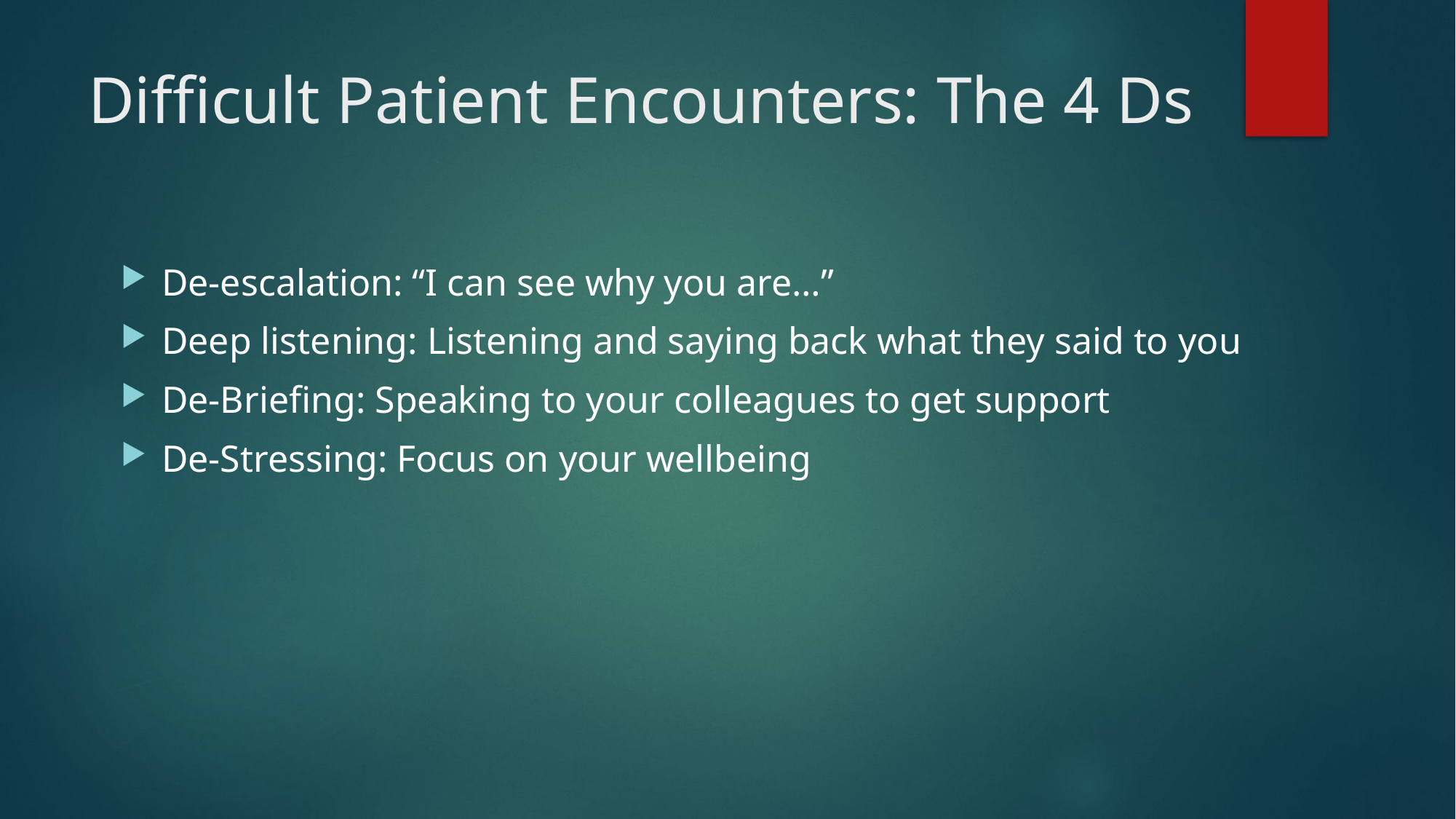

# Difficult Patient Encounters: The 4 Ds
De-escalation: “I can see why you are…”
Deep listening: Listening and saying back what they said to you
De-Briefing: Speaking to your colleagues to get support
De-Stressing: Focus on your wellbeing
